# IL-8, IL-10, TGF-**β**, and GCSF Levels Were Increased in Severe Persistent Allergic Asthma Patients with the Anti-IgE Treatment

**DOI:** 10.1155/2012/720976

**Published:** 2012-12-19

**Authors:** Arzu D. Yalcin, Atil Bisgin, Reginald M. Gorczynski

**Affiliations:** ^1^Allergy and Clinical Immunology Unit, Department of Internal Medicine, Antalya Training and Research Hospital, 07070 Antalya, Turkey; ^2^Department of Clinical and Experimental Medicine, Faculty of Health Sciences, Linköping University, 58185 Linköping, Sweden; ^3^Department of Medical Genetics, Faculty of Medicine, Cukurova University, 01330 Adana, Turkey; ^4^Division of Cellular and Molecular Biology, Toronto Hospital, University Health Network, Toronto, ON, Canada M5G 2C4

## Abstract

*Background*. Allergic asthma is showed an increase in Th2-cytokine and IgE levels and an accumulation activation of Th2 cells, eosinophils and mast cells. However, recent studies focused on cell-based mechanisms for the pathogenesis of allergic asthma. *Objectives*. In this study, we compare the anti-IgE treatment modality in the dynamics of immune system cytokine levels in severe persistent asthma (SPA) patients who had no other any allergic disease, newly diagnosed allergic asthma patients and healthy volunteers. *Study Design*. The study population consisted of 14 SPA patients, 14 newly diagnosed allergic asthma patients and 14 healthy volunteers included as controls. Cytokine levels were measured. Total and specific IgE levels of anti-IgE monoclonal antibody treated patients, serum high-sensitivity C-reactive protein (hsCRP) levels, FEV1/FVC rates and asthma control test (ACT) were measured for the clinical follow-up. *Results*. We observed that SPA patients presented increasing levels of IL-8, IL-10, TGF-**β** and GCSF during the anti-IgE treatment in period of sampling times at 4 months and 18 months. However this increase was not correlated neither with serum hsCRP levels nor FEV1/FVC rates. *Conclusions*. Our study gives a different perspective for the SPA and anti-IgE immunotherapy efficacy at the cell cytokine-linked step.

## 1. Introduction

Asthma is the most common serious chronic lung disease that affects people of all ages with evidence for a growing prevalence in industrialized as well as in developing countries [1, 2]. With airway hyper-responsiveness being the physiological hallmark of asthma, it is also characterized by chronic inflammation of the respiratory tract, allergen-specific IgE production, infiltration of eosinophils, the recruitment of T cells into the airways, and alterations in the fine balance between type 1 helper T lymphocytes (Th1) and type 2 helper T lymphocytes (Th2) responses towards Th2 bias [3, 4].

Th2 cells secrete a panel of cytokines with several overlapping functions including Interleukin-4 (IL-4), IL-5, IL-13, and granulocyte-macrophage colony stimulating factor (GM-CSF). By mediating differentiation of the Th2 subpopulation and eosinophils, as well as modulating B-cell proliferation and IgE switching, the Th2 cytokines are thought to play a prominent role in asthma [5, 6]. The sentinel Th1 cytokine, interferon gamma (IFN*γ*), and IL-12 reciprocally stimulate their production and function during cell-mediated immunity and development of naïve T lymphocytes into Th1 cells. Evidence suggests a contributory role of Th1 cells and their cytokines in asthmatic inflammation and airway hyper-responsiveness [7, 8]. The T cell subset of regulatory T cells (Treg) acts by expressing immunosuppressive cytokines, such as IL-10, of which impaired production has been reported in asthmatic patients [9]. Moreover, the lymphocyte lineage Th17 is increased in inflamed airways and characterized by the production of IL-17 [10, 11]. This proinflammatory cytokine is capable of causing the release of other proinflammatory cytokines, such as IL-8, tumor necrosis factor alpha (TNF*α*), and GM-CSF, which have been associated with asthma in murine models, in humans or with disease severity [12–18].

Asthma—probably the most heterogeneous lung disease—classification is based on severity and there is no universally accepted utility in diagnosis of asthma and certain subtypes. Current Global Initiative for Asthma (GINA) guidelines emphasize the need to evaluate asthma control to guide asthma management decisions. The Asthma Control Test (ACT) questionnaire—a simple, self-administered, and rapidly completed assessment tool—is also appropriate to assess the patients and has an advantage of can be applied at all levels of healthcare. Symptoms in the most severe form of asthma, also called ‘‘severe persistent allergic asthma”, are thought to be precipitated by allergens. In addition to allergens, environmental factors or infectious pathogens often trigger epithelial stress and altered innate immunity that induce different types of inflammation, thereby resulting in the heterogeneous forms of asthma.

Most recent treatment modality—anti-IgE therapy—was developed for severe allergic asthma. Anti-IgE therapy affects by lowering free IgE and leading to downregulation of high-affinity IgE receptors on circulating basophils and mast cells. So that the early and late phase responses to inhaled allergens will be attenuated [19–22].

This study surveyed the levels of chosen serum IL-8, IL-17, TGF-*β*, and GCSF of the allergic asthma patients treated with anti-IgE therapy to investigate their roles in the pathogenesis of disease perpetuation, and anti-IgE therapy's impact on them.

## 2. Materials and Methods

### 2.1. Patients Samples

Twenty eight allergic asthma—allergic rhinitis patients were included in the study and divided into two groups according to the severity. In the first group there were 14 patients of 5 male and 9 female, whom were suffering from severe persistent allergic asthma—allergic rhinitis and underwent anti-IgE therapy for 18 months within the product label (omalizumab) every 2 weeks. Assessment of clinical changes and adverse effects were evaluated at each bimonthly patient visit including vital signs, full physical examination, details of any allergy incidents, total and specific IgE levels, serum high-sensitivity C-reactive protein levels, pulmonary function test (FEV1/FVC rates), and asthma control test (ACT) (Quality Metric Incorp.). A spirometry was performed at each visit (once or twice a month depending on the patient's visit schedule). Reference values for the Mediterranean population were used [23]. Need for the steroid therapy and doses they were using were given in Tables 1 and 2. Blood samples were taken during these followups first in the time of diagnosis (Group IA), 4 months after the anti-IgE therapy (Group IB), and at the 18th month of treatment during the remission (Group IC).

The other patients group included the newly diagnosed allergic asthma-allergic rhinitis patients (non-severe) (Group II).

The healthy volunteers (group III, *n* = 14) had no history of allergy/atopy, family atopy, cardiac and pulmonary diseases or smoking.

The study was approved by the local ethics committee, and written consent was obtained from all patients and healthy volunteers.

### 2.2. Treatment Control

Patients were asked to describe their asthma treatment at each outpatient visit, and the total monthly oral corticosteroid dose was recorded. In the case of exacerbation, patients were asked to come to the hospital, if possible to the outpatient center at our pulmonary service during business hours rather than the emergency room (ER) in order to facilitate treatment control. Nonetheless, data for patients who came to the ER and discharge treatment were recovered, since the clinical histories at the hospital are computerized.

### 2.3. Skin Prick Test (SPT)

Skin prick tests on the forearm were performed in all patients using standardized latex extract containing high ammonia natural rubber latex, and a full set of 35 common and 35 food allergens. In addition, venom SPT was performed on one patient based on the subject's clinical history. SPTs were performed by skilled nursing personnel. Positive tests were counted as wheals of 3 mm in diameter after 20 minutes. Tests were compared with positive histamine controls and negative saline controls. Commercial extracts used were manufactured by Allergopharma (Germany). No intradermal tests were performed.

### 2.4. Treatment Protocol

Best Standard Care (BSC) following the recommendations of the GINA included inhaled corticosteroids (fluticasone 500 mg bid), inhaled long-acting beta-agonists (LABA) (salmeterol 50 mg bid), and oral methyl-prednisolone. Prior to starting omalizumab treatment, patients underwent a run-in period of at least 18 months. The protocol followed for decreasing oral steroid administration was as follows; the daily dose was decreased by 2 mg/day; if the patient remained stable, at the end of the two weeks the daily dose was decreased by a further 2 mg for the following weeks. Steroid dose was then increased to the previous level and the process was repeated.

### 2.5. Experimental Procedures

Concentrations of IL-8, IL-10, IL-17, TNF-*α*, TGF-*β*, and GCSF in the serum samples were quantified using ELISA kits. The assays were performed according to the recommendations of the manufacturer using standard curve for every cytokine. The results were reported as means of duplicate measurements.

Total and specific IgE levels were enumerated by fluoroenzyme immunoassay (ImmunoCAP—FEIA) using an ImmunoCAP (Pharmacia, Uppsala, Sweden) kit. Values above 100 kU/L and 0.35 kU/L for total and specific IgE levels were considered abnormal.

Serum hs-CRP levels were measured using a hs-CRP assay (Behring Latex-Enhanced using the Behring Nephelometer BN-100; Behring Diagnostics, Westwood, MA, USA). The sensitivity of the assay ranged 0.04–5.0 mg/L.

### 2.6. Statistical Analysis

All the data were analyzed by using student *t*-test with the statistical package for the Social Sciences 13.0 software for Windows (SPSS Inc., Chicago, III). A *P* value less than 0.05 was considered to be statistically significant. GraphPad Prism version 5 (La Jolla, CA, USA) were used to plot the data and perform correlation analyses. All correlation analyses used Spearman's Rho tests.

## 3. Results

Main demographic and clinical characteristics of study participants were summarized in Tables 1 and 2. Clinical data from the patients during the treatment with anti-IgE, indicated the beneficial effects on symptoms and perceived quality of life without any exacerbation, as well as a reduction in unscheduled healthcare visits.

**Figure 1:**
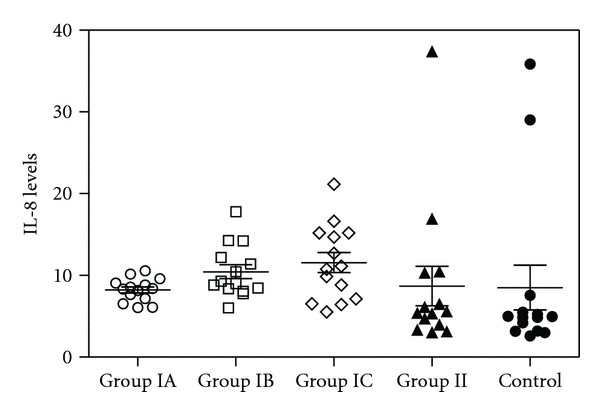
Plot graphic of serum IL-8 concentrations in all groups. The numbers of samples of the all groups are 14 for each. Group IA: severe persistent asthma patients before the treatment. Group IB: 4 months after the anti-IgE therapy, severe persistent asthma patients. Group IC: 18 months after the anti-IgE therapy, severe persistent asthma patients. Group II: newly diagnosed controlled allergic asthma patients. Group III: healthy individuals as control. *P* values were as below: Group IA versus IB: *P* = 0.02, Group IA versus IC: *P* = 0.019, Group IA versus II: *P* = 0.42, Group IA versus Control: *P* = 0.46, Group IB versus IC: *P* = 0.27, Group IB versus Control: *P* = 0.25, Group IC versus Control: *P* = 0.16, and Group II versus Control: *P* = 0.48.

**Figure 2:**
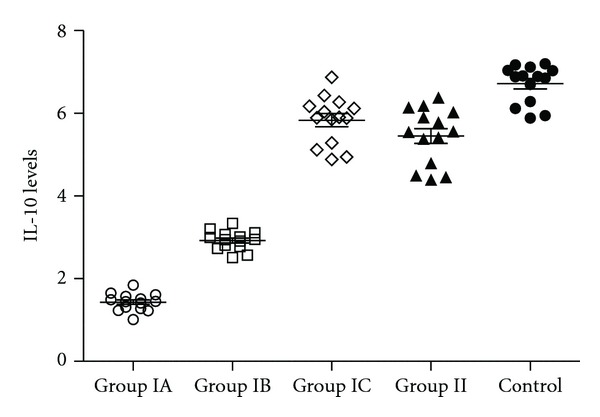
Serum IL-10 levels of all study groups. The numbers of samples of the all groups are 14 for each. Group IA: severe persistent asthma patients before the treatment. Group IB: 4 months after the anti-IgE therapy, severe persistent asthma patients. Group IC: 18 months after the anti-IgE therapy, severe persistent asthma patients. Group II: newly diagnosed controlled allergic asthma patients. Group III: healthy individuals as control. *P* values were as below: Group IA versus IB: *P* < 0.0001, Group IA versus IC: *P* < 0.0001, Group IA versus II: *P* < 0.0001, Group IA versus Control: *P* < 0.0001, Group IB versus IC: *P* = 0.0024, Group IB versus Control: *P* < 0.0001, Group IC versus Control: *P* = 0.0018, and Group II versus Control: *P* < 0.0001.

**Figure 3:**
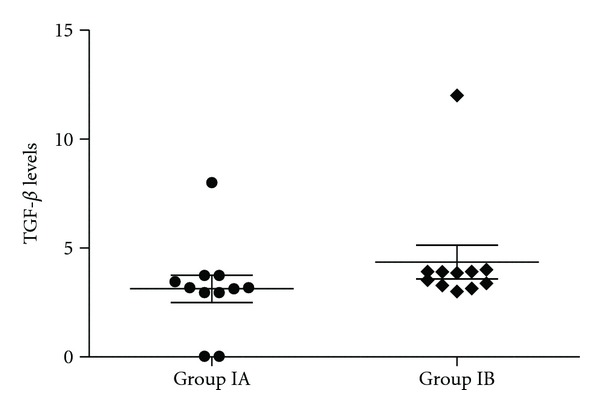
TGF-*β* levels of severe persistent asthma patients before and 4 months after the anti-IgE therapy (*P* = 0.013).

**Figure 4:**
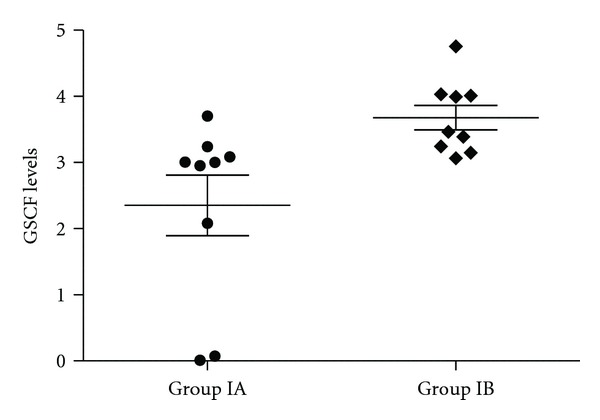
The concentration of GSCF in severe persistent asthma patients in group IA and IB (*P* = 0.009).

**Figure 5:**
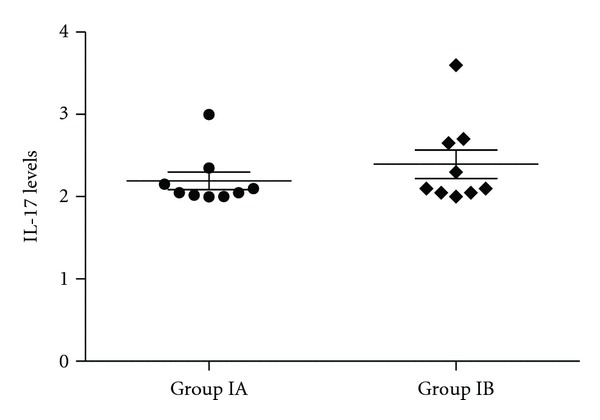
Serum IL-17 levels of severe persistent asthma patients were shown in dot-plot graph (*P* = 0.17).

In Table 2, healthy and asthmatic patients are compared as well as healthy and severe persistent asthma patients. Except of IL-8, IL-10, TGF-*β*, and GCSF in the anti-IgE treated severe persistent asthma patients group, none of the cytokine concentrations in serum differ significantly between healthy and severe persistent asthma diseased patients (Figures 1, 2, 3, 4, and 5—only the significant data with *P* values were given as in figures). These values were also increased during the anti-IgE therapy and this difference is significant between the fourth/eighteenth month of anti-IgE therapy and control group and newly diagnosed allergic asthma patients. In contrast, the levels of IL-8, TGF-*β*, and GCSF did not differ between newly diagnosed allergic asthma patients, non-treated severe persistent asthma patients and control. Moreover, the mean serum IL-10 levels were lower than the control group in newly diagnosed allergic asthma patients and non-treated severe persistent asthma patients. Furthermore, IL-17 levels did not change between any groups and are not associated with any clinical parameters of asthma.

**Figure 6:**
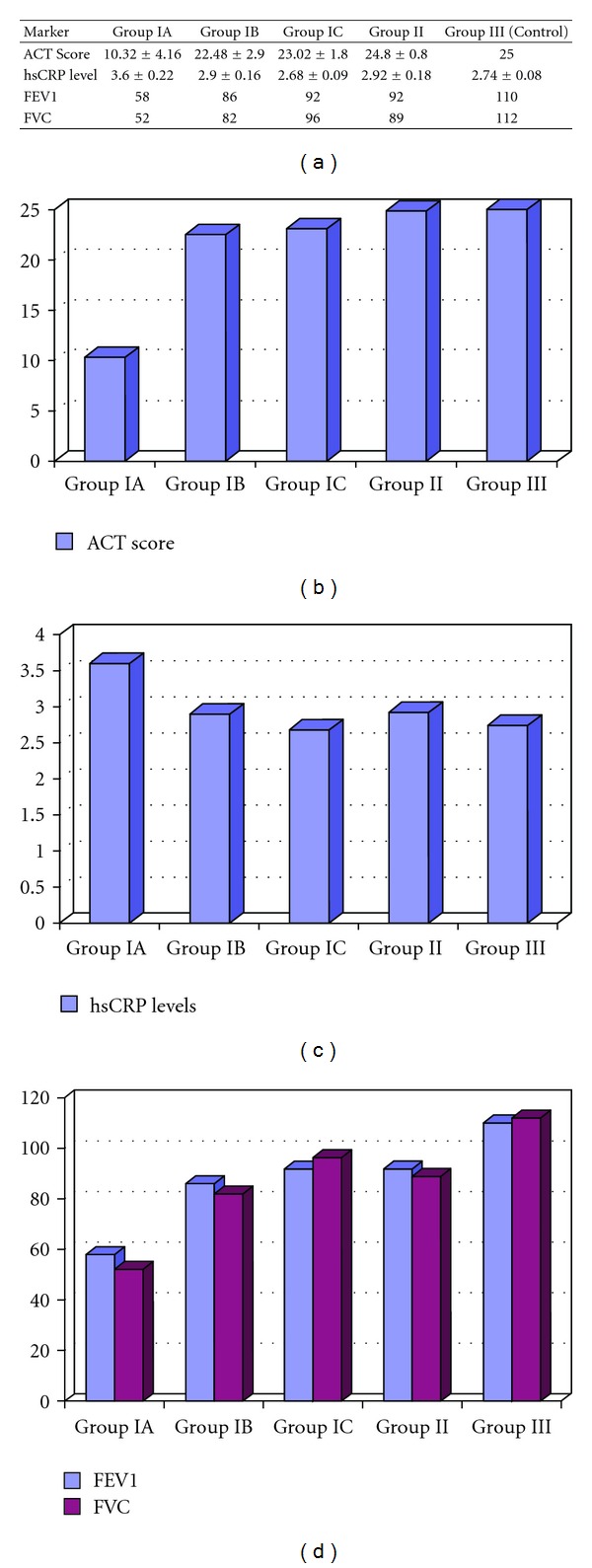
Clinical follow-up markers; FEV1/FVC rate, serum hsCRP levels, and ACT score of groups. ACT score was significantly increased similar to the controls after anti-IgE therapy (Group IA versus IB: *P* = 0.005). HsCRP levels were significantly decreased to the levels of controls after the treatment (Group IA versus IB: *P* = 0.04 and Group IA versus IC: *P* = 0.02). FEV1 and FVC values also significantly increased by the anti-IgE treatment depending on the time of therapy (FEV1: Group IA versus IB: *P* = 0.02 and Group IA versus IC: *P* = 0.01 and FVC: Group IA versus IB: *P* = 0.01 and Group IA versus IC: *P* = 0.008). Values are presented as mean ± standard deviation (SD). Additional bar graphs used to compare data are given.

Results of serum cytokine measurements of the severe persistent asthma patients and distinct clinical parameters were then further analyzed. However, this increase was not correlated neither with any of the clinical follow-up markers and serum hsCRP levels. Even though, ACT score of the patients and serum hsCRP levels together with FEV1/FVC status were significantly different between two patient groups (see Figures 6(a), 6(b), 6(c), and 6(d)).

Prick tests were in all patients in Group I and Group II were detected in mite and grass allergy. These results correlated with specific IgE. The study subjects' baseline characteristics are shown in Tables 1 and 2. The mean IgE levels were as follow: (Group IA: 551.99 IU/mL; Group II: 144.25 IU/mL and Group III: 38.62 IU/mL).

## 4. Discussion

Multiple pathophysiological effects have been associated with imbalanced T cell activation and the presence or absence of distinct immune mediators in asthma patients [18]. Thus there has developed an increased interest in the role of cytokines and chemokines for diagnosis and therapy. The present study aimed at understanding the pattern of expression of several circulating cytokines in asthmatic individuals. To assess the potential value of these cytokines as biomarker for asthma, or certain asthma phenotypes, or prediction of anti-IgE therapy efficacy, we compared serum levels of these asthma associated mediators in two groups: patients with severe persistent asthma treated with anti-IgE therapy, and newly diagnosed allergic asthma patients. Anti-IgE therapy with omalizumab reduces serum levels of free IgE and downregulates expression of IgE receptors (Fc epsilonRI) on mast cells and basophils. In the airways of patients with mild allergic asthma, omalizumab reduces Fc epsilon RI+ and IgE+ cells and causes a profound reduction in tissue eosinophilia, together with reductions in submucosal T-cell and B-cell numbers. Omalizumab decreases Fc epsilonRI expression on circulating dendritic cells, which might lead to a reduction in allergen presentation, T(h)2 cell activation, and proliferation. And our result of no difference between anti-IgE treated patients and the control group emphasize the fundamental importance of anti-inflammatory effects of omalizumab and IgE in allergic inflammation. A number of authors have studied serum cytokines in asthmatic individuals [24–28]. However, no study focused on the relation between these cytokine levels and anti-IgE therapy.

Distinct type of asthma is related to neutrophilic inflammation. However, there is still a multicellular process leads to multicellular inflammation in the pathogenesis of asthma [29]. IL-8 takes role in the activation of neutrophils and is a potent chemoattractant of neutrophils during the airway inflammation [30]. There is also growing evidence that IL-17 is involved in the pathogenesis of asthma. IL-17 orchestrates the neutrophilic influx into the airways and also enhances T-helper 2 (Th2) cell-mediated eosinophilic airway inflammation in asthma [31, 32]. Moreover eosinophils are also a central feature in asthma and are very prominent cells. And GM-CSF promotes eosinophil activation and survival [33]. Eosinophils are also thought to be an important source of the potent pro-fibrotic cytokine TGF-*β*, although numerous other cells types including platelets, fibroblasts, smooth muscle and epithelial cells can also produce TGF-*β*. However, the precise role of eosinophil-derived TGF-*β* in airway remodeling is complicated and related to both eosinophils and mast cells [34–36].

Researchers therefore should keep in mind that the change in cytokine levels in the context of asthma, inflammation, and within different treatment modalities, and discuss the therapeutic potential of various strategies targeting cytokines for asthma that might have been applied as a therapeutic approach.

In our study for this purpose we evaluated the cytokine levels of different T cell sub-types. However, no differences were observed in IL-8 levels between healthy and diseased individuals before anti-IgE therapy. IL-10 levels were higher in treated patients and healthy individuals than the newly diagnosed patients as it was previously reported that inhaled corticosteroid therapy restores the reduced IL-10 release [37] IL-17 levels did not change during the anti-IgE therapy in severe persistent asthma patients. In contrast, IL-8, IL-10, TGF-*β*, and GSCF patterns showed a statistically significant difference in patients before/after therapy, suggesting a value in monitoring circulating cytokine levels in severe persistent asthma patients receiving anti-IgE therapy that also indicates that anti-IgE therapy provides clinical benefits. In our study, the levels of TNF-*α* were also investigated because of its important role in the bronchus allergic inflammation. However, there was no significant difference in the level between groups (data not shown).

It has been suggested that asthma is not necessarily associated with changes of serum cytokines [1]. However, this controversy may be in part at least explained by the heterogeneity of the overall asthmatic patient population. Asthma patients referred to our clinic in this study were divided into two subgroups; group I patients with severe persistent asthma for periods ranging from 3 to 7 years, and group II subjects who were diagnosed as allergic asthma with a history ranging from 6 to 27 years. Group I patients had been receiving anti-IgE therapy while group II received inhalant steroids therapy and had been classified as controlled allergic asthma subjects for 1 to 3 years. According to our previous experiences of anti-IgE therapy in clinical use, its indications and our studies on, the clinical effect begins at the third month of treatment [38, 39]. And no other exacerbations had seen on the patients after then. So that might be in the relation of alterations in cytokine expressions profiles and clinical symptoms during the omalizumab treatment.

We also evaluated serum cytokine levels in relation to clinical parameters, including total and specific IgE, asthma onset, pulmonary function tests, hsCRP level, and ACT. There was no clear pattern in the expression levels of circulating cytokines and clinical parameters of asthma. In this regard, our results are in accord with previous studies that indicate that serum cytokine levels reflecting activity of Th1, Th2, and Th17 cells and clinical symptoms are independent of one another [24, 28, 40–42]. Note that the hsCRP levels and FEV1/FVC rates were different from healthy individuals in both group I and group II patients, reflecting the clinical manifestations of asthma.

IL-8 is a pro-neutrophilic chemokine that is secreted by various cell types. It is thought to play an important role in asthma, with levels correlated with the severity of disease [14, 16, 18]. We found IL-8 levels increased along with those of IL-10 the immune regulatory and anti-inflammatory cytokine and TGF-*β* and GSCF in severe persistent asthma patients who were receiving anti-IgE therapy. Anti-IgE (omalizumab) treatment attenuates both the early- and late-phase responses to inhaled allergens in patients with asthma [19]. Further anti-inflammatory effects, including changes in interleukin levels, have been observed and postulated to contribute to the clinical efficacy of omalizumab treatment [20, 21]. Other studies in severe persistent allergic asthma patients receiving omalizumab therapy have focused on modulation of serum soluble TNF-related apoptosis-inducing ligand, total antioxidant capacity, hydrogen peroxide, malondialdehyde and total nitric oxide concentrations, and ceruloplasmin oxidase activity measurements, as markers of the efficacy of anti-IgE treatment modality [43–46]. Our data add IL-8, IL-10, TGF-*β*, and GCSF to this list.

Both local and systemic inflammation is associated with pathogenesis in asthma [47, 48]. To assess systemic inflammation, we monitored serum levels of CRP in patients. Because of possible confounding effects on CRP levels, subjects with kidney disease, heart disease, liver disease, diabetes mellitus, cancer, obesity, smoking history, and autoimmune disease were excluded from our study. No correlation was observed between levels of any of the cytokines measured, clinical outcome, and serum hsCRP concentrations.

In conclusion, the present study documents evidence for altered patterns in serum cytokines in severe persistent asthma patients following anti-IgE therapy. However, the basal serum cytokine profiles excluding the IL-10, patterns were not different between healthy and asthmatic individuals, regardless of whether the latter were newly diagnosed allergic asthma or non-treated severe persistent asthmatic patients. We believe this study provides a novel perspective on the mechanism of action of anti-IgE immunotherapy in severe persistent asthma patients and inflammatory mediators in defining clinical benefits.

## Figures and Tables

**Table 1:** Demographics of severe persistent asthma patients (Group I).

Patient	Age (y) and Sex	Prick Test Positivity	Number of Injection	Injection Dose of Omalizumab	Inhalant Steroid Doses Pre-omalizumab	Oral Steroid Doses Pre-omalizumab
1	62 male	Grass, tree, mold, mite	30	375 mg q. 2 weeks	600 *μ*g	6 mg
2	39 male	Grass, mite, cockroach	28	225 mg q. 2 weeks	500 *μ*g	8 mg
3	50 male	Wheat, Mite, tree	31	300 mg q. 2 weeks	400 *μ*g	0
4	19 female	Mold, mite, dog epithelia	27	225 mg q. 2 weeks	500 *μ*g	0
5	18 female	Grass, wheat, tree, mold, mite, cockroach, kiwi and orange, cat epithelia	29	300 mg q. 2 weeks	500 *μ*g	0
6	57 female	Grass, tree, mite	33	300 mg q. 2 weeks	600 *μ*g	6 mg
7	50 female	Grass, wheat, tree, mold, mite, cockroach, tomato, eggplant, strawberry, dog and cat epithelia	34	300 mg q. 2 weeks	800 *μ*g	8 mg
8	34 female	Grass, tree, mite	29	300 mg q. 2 weeks	600 *μ*g	6 mg
9	42 female	Grass, wheat, tree, mold, mite, cockroach, honeybee, dog and cat epithelia	31	300 mg q. 2 weeks	1200 *μ*g	12 mg
10	59 male	Grass, wheat, tree, mold, mite, cockroach, shrimp, perch, egg and latex	32	300 mg q. 2 weeks	1000 *μ*g	10 mg
11	49 female	Grass, tree, mite, dog epithelia	28	300 mg q. 2 weeks	800 *μ*g	8 mg
12	37 female	Mold, mite, cockroach	34	300 mg q. 2 weeks	400 *μ*g	0
13	52 male	Mold, mite, dog epithelia	21	300 mg q. 2 weeks	600 *μ*g	6 mg
14	48 female	Grass, wheat, tree, mite, cockroach	23	225 mg q. 2 weeks	400 *μ*g	0

**Table 2:** Demographics of controlled allergic asthma patients (Group II) and control group (Group III).

Patient	Age (y) and Gender Group II	Inhalant Steroid Doses Group II	Prick Test Positivity of Group II	Age (y) and Gender Group III
1	58 male	200 *μ*g	Grass, mite, cockroach	58 male
2	42 male	200 *μ*g	Grass, mite, cockroach	39 male
3	54 male	100 *μ*g	Wheat, mite, grass	55 male
4	22 female	100 *μ*g	Mold, mite	21 female
5	20 female	200 *μ*g	Grass, wheat, tree, mold, cat epithelia	19 female
6	62 female	100 *μ*g	Grass, tree, mite	58 female
7	49 female	100 *μ*g	Grass, wheat, mold, mite, dog and cat epithelia	50 female
8	40 female	200 *μ*g	Grass, cockroach, mite	38 female
9	43 female	100 *μ*g	Grass, tree, mold, mite, cat epithelia	43 female
10	59 male	100 *μ*g	Grass, tree, mold, mite	61 male
11	49 female	100 *μ*g	Mite, dog epithelia, tree	50 female
12	39 female	100 *μ*g	Mold, mite, cockroach	42 female
13	52 male	200 *μ*g	Mold, mite, cockroach, dog and cat epithelia	53 male
14	48 female	100 *μ*g	Grass, wheat, tree, mite, cockroach	50 female

## References

[1] Anderson GP (2008). Endotyping asthma: new insights into key pathogenic mechanisms in a complex, heterogeneous disease. *The Lancet*.

[2] Lotvall J, Akdis C, Bacharier LB (2011). Asthma endotypes: a new approach to classification of disease entities within the asthma syndrome. *American Academy of Allergy, Asthma, and Immunology*.

[3] Larche M, Robinson DS, Kay AB (2003). The role of T lymphocytes in the pathogenesis of asthma. *The Journal of Allergy and Clinical Immunology*.

[4] Epstein MM (2006). Targeting memory Th2 cells for the treatment of allergic asthma. *Pharmacology & Therapeutics*.

[5] Robinson DS, Hamid Q, Ying S (1992). Predominant T(H2)-like bronchoalveolar T-lymphocyte population in atopic asthma. *The New England Journal of Medicine*.

[6] Wills-Karp M, Finkelman FD (2008). Untangling the complex web of IL-4- and IL-13-mediated signaling pathways. *Science Signaling*.

[7] Cooper AM, Khader SA (2007). IL-12p40: an inherently agonistic cytokine. *Trends in Immunology*.

[8] Kumar RK, Webb DC, Herbert C, Foster PS (2006). Interferon-*γ* as a possible target in chronic asthma. *Inflammation and Allergy*.

[9] John M, Lim S, Seybold J (1998). Inhaled corticosteroids increase interleukin-10 but reduce macrophage inflammatory protein-1*α*, granulocyte-macrophage colony-stimulating factor, and interferon-*γ* release from alveolar macrophages in asthma. *American Journal of Respiratory and Critical Care Medicine*.

[10] Pene J, Chevalier S, Preisser L (2008). Chronically inflamed human tissues are infiltrated by highly differentiated Th17 lymphocytes. *The Journal of Immunology*.

[11] Wang YH, Voo KS, Liu B (2010). A novel subset of CD4^+^ TH2 memory/ effector cells that produce inflammatory IL-17 cytokine and promote the exacerbation of chronic allergic asthma. *Journal of Experimental Medicine*.

[12] Yamashita N, Tashimo H, Ishida H (2002). Attenuation of airway hyperresponsiveness in a murine asthma model by neutralization of granulocyte-macrophage colony-stimulating factor (GM-CSF). *Cellular Immunology*.

[13] Dente FL, Carnevali S, Bartoli ML (2006). Profiles of proinflammatory cytokines in sputum from different groups of severe asthmatic patients. *Annals of Allergy, Asthma and Immunology*.

[14] Shute JK, Vrugt B, Lindley IJD (1997). Free and complexed interleukin-8 in blood and bronchial mucosa in asthma. *American Journal of Respiratory and Critical Care Medicine*.

[15] Berry MA, Hargadon B, Shelley M (2006). Evidence of a role of tumor necrosis factor alpha in refractory asthma. *The New England Journal of Medicine*.

[16] Jatakanon A, Uasuf C, Maziak W, Lim S, Chung KF, Barnes PJ (1999). Neutrophilic inflammation in severe persistent asthma. *American Journal of Respiratory and Critical Care Medicine*.

[17] Saha SK, Doe C, Mistry V (2009). Granulocyte-macrophage colony-stimulating factor expression in induced sputum and bronchial mucosa in asthma and COPD. *Thorax*.

[18] Silvestri M, Bontempelli M, Giacomelli M (2006). High serum levels of tumour necrosis factor-*α* and interleukin-8 in severe asthma: markers of systemic inflammation?. *Clinical and Experimental Allergy*.

[19] Fahy JV, Fleming HE, Wong HH (1997). The effect of an anti-IgE monoclonal antibody on the early- and late- phase responses to allergen inhalation in asthmatic subjects. *American Journal of Respiratory and Critical Care Medicine*.

[20] Noga O, Hanf G, Brachmann I (2006). Effect of omalizumab treatment on peripheral eosinophil and T-lymphocyte function in patients with allergic asthma. *Journal of Allergy and Clinical Immunology*.

[21] Hanf G, Brachmann I, Kleine-Tebbe J (2006). Omalizumab decreased IgE-release and induced changes in cellular immunity in patients with allergic asthma. *Allergy*.

[22] Yalcin AD, Bisgin A, Cetinkaya R, Gumuslu S Clinical efficacy of omalizumab in severe persistent asthma and co-morbid conditions.

[23] Roca J, Sanchis J, Agusti-Vidal A (1986). Spirometric reference values from a Mediterranean population. *Clinical Respiratory Physiology*.

[24] Hollander C, Sitkauskiene B, Sakalauskas R, Westin U, Janciauskiene SM (2007). Serum and bronchial lavage fluid concentrations of IL-8, SLPI, sCD14 and sICAM-1 in patients with COPD and asthma. *Respiratory Medicine*.

[25] Krogulska A, Wasowska-Królikowska K, Polakowska E, Chrul S (2009). Cytokine profile in children with asthma undergoing food challenges. *Journal of Investigational Allergology and Clinical Immunology*.

[26] Litonjua AA, Sparrow D, Guevarra L, O’Connor GT, Weiss ST, Tollerud DJ (2003). Serum interferon-*γ* is associated with longitudinal decline in lung function among asthmatic patients: the Normative Aging Study. *Annals of Allergy, Asthma and Immunology*.

[27] Nakamura H, Weiss ST, Israel E, Luster AD, Drazen JM, Lilly CM (1999). Eotaxin and impaired lung function in asthma. *American Journal of Respiratory and Critical Care Medicine*.

[28] Tateno H, Nakamura H, Minematsu N (2004). Plasma eotaxin level and severity of asthma treated with corticosteroid. *Respiratory Medicine*.

[29] Holgate ST (2007). Epithelium dysfunction in asthma. *Journal of Allergy and Clinical Immunology*.

[30] Nocker RET, Schoonbrood DFM, van de Graaf EA (1996). Interleukin-8 in airway inflammation in patients with asthma and chronic obstructive pulmonary disease. *International Archives of Allergy and Immunology*.

[31] Hsu HC, Yang PA, Wang J (2008). Interleukin 17-producing T helper cells and interleukin 17 orchestrate autoreactive germinal center development in autoimmune BXD2 mice. *Nature Immunology*.

[32] Sun YC, Zhou QT, Yao WZ (2005). Sputum interleukin-17 is increased and associated with airway neutrophilia in patients with severe asthma. *Chinese Medical Journal*.

[33] Trivedi SG, Lloyd CM (2007). Eosinophils in the pathogenesis of allergic airways disease. *Cellular and Molecular Life Sciences*.

[34] Minshall EM, Leung DYM, Martin RJ (1997). Eosinophil-associated TGF-*β*1 mRNA expression and airways fibrosis in bronchial Asthma. *American Journal of Respiratory Cell and Molecular Biology*.

[35] Cho JY, Miller M, Baek KJ (2004). Inhibition of airway remodeling in IL-5-deficient mice. *Journal of Clinical Investigation*.

[36] Brightling CE, Bradding P, Symon FA, Holgate ST, Wardlaw AJ, Pavord ID (2002). Mast-cell infiltration of airway smooth muscle in asthma. *The New England Journal of Medicine*.

[37] Chung F (2001). Anti-inflammatory cytokines in asthma and allergy: Interleukin-10, interleukin-12, interferon-*γ*. *Mediators of Inflammation*.

[38] Yalcin AD (2012). Bisgin A The relation of sTRAIL levels and quality of life in severe persistent allergic asthma patients using omalizumab. *Medical Science Monitor*.

[39] Yalcin AD, Bisgin A Omalizumab: anti-IgE therapy in severe allergic conditions. *Allergy & Therapy Journals*.

[40] Corrigan CJ, Kay AB (1990). CD4 T-lymphocyte activation in acute severe asthma. Relationship to disease severity and atopic status. *American Review of Respiratory Disease*.

[41] Saito H, Hayakawa T, Mita H, Yui Y, Shida T (1988). Augmentation of leukotriene C4 production by gamma interferon in leukocytes challenged with an allergen. *International Archives of Allergy and Applied Immunology*.

[42] Friebe A, Volk HD (2008). Stability of tumor necrosis factor *α*, interleukin 6, and interleukin 8 in blood samples of patients with systemic immune activation. *Archives of Pathology and Laboratory Medicine*.

[43] Yalcin AD, Gorczynski RM, Parlak GE (2012). Total antioxidant capacity, hydrogen peroxide, malondialdehyde and total nitric oxide concentrations in patients with severe persistent allergic asthma: its relation to omalizumab treatment. *Clinical Laboratory*.

[44] Yalcin AD, Bisgin A, Kargi A, Gorczynski RM (2012). Serum soluble TRAIL levels in patients with severe persistent allergic asthma: its relation to Omalizumab treatment. *Medical Science Monitor*.

[45] Yalcin AD, Gumuslu S, Parlak GE (2012). Systemic levels of ceruloplasmin oxidase activity in allergic asthma and allergic rhinitis. *Immunopharmacol Immunotoxicol*.

[46] Bates CA, Silkoff PE (2003). Exhaled nitric oxide in asthma: from bench to bedside. *Journal of Allergy and Clinical Immunology*.

[47] Pepys MB, Baltz ML (1983). Acute phase proteins with special reference to C-reactive protein and related proteins (pentaxins) and serum amyloid A protein. *Advances in Immunology*.

[48] Jousilahti P, Salomaa V, Hakala K, Rasi V, Vahtera E, Palosuo T (2002). The association of sensitive systemic inflammation markers with bronchial asthma. *Annals of Allergy, Asthma and Immunology*.

